# Endoscopic Iliotibial Band Release is an Effective Treatment for External Snapping Hip Syndrome: A Case Series

**DOI:** 10.7759/cureus.91048

**Published:** 2025-08-26

**Authors:** Maria Tsatlidou, Alexandros P Tzaveas, Theofylaktos Kyriakidis, Michalis Iosifidis

**Affiliations:** 1 Third Orthopaedic Department, European Interbalkan Medical Center, Thessaloniki, GRC; 2 Orthopaedics Department, OrthoBiology Surgery Center, Thessaloniki, GRC

**Keywords:** endoscopic iliotibial band release, external snapping hip syndrome, hip arthroscopy, iliotibial band lengthening, snapping hip

## Abstract

Introduction

External snapping hip syndrome (ESHS) is characterized by a snapping sensation and potential pain, particularly during hip flexion and extension. It affects the young and athletic population, and its principal cause is a tight iliotibial band (ITB) sliding over the greater trochanter (GT). Multiple surgical interventions have been proposed when conservative treatment fails. However, the open techniques are associated with slower mobilization, large scars, and relatively high complication rate compared to the endoscopic procedures.

Material and methods

This study retrospectively examines the clinical outcomes of endoscopic ITB release using a modified Ilizaliturri method in seven patients diagnosed with ESHS, following unsuccessful conservative management. The endoscopic technique involved a cross-shaped incision on the ITB, allowing for effective release. The modified Harris Hip Score (MHHS) and Hip Outcome Score (HOS) were utilized to measure improvement.

Results

Six patients experienced uneventful recoveries, with one minor complication that resolved spontaneously. In a mean follow-up of 25 months, there was no recurrence of symptoms. Statistical analysis revealed significant improvement of both MHHS and HOS.

Conclusion

The present study demonstrates that this endoscopic procedure is a safe and effective option for managing ESHS, presenting favorable outcomes with minimal complications. Further research with larger sample sizes is necessary to establish comprehensive comparisons between open and endoscopic techniques.

## Introduction

The Iliotibial band (ITB) is a structure that acts as a stabilizer of the hip during walking and abduction. As its proximal origin is the tensor fascia lata anteriorly and gluteus maximus posteriorly, the ITB is sliding over the greater trochanter (GT) during contraction of the above muscles, when hip flexion and extension are performed [1]. In cases of taut ITB complex that may involve the gluteus maximus insertion, a tight fascia lata, or even an enlarged greater trochanter, a snapping sensation might occur during movement. This entity, known as “external snapping hip” or lateral “coxa saltans” was first described by Perrin in 1859 [2-4], and it is reported to affect about ten percent of the general population with a higher prevalence among athletic individuals, especially engaged in sports that require wide range of motion (ROM) of the hip, such as runners, football players, and dancers [1-4]. Although snapping is generally not painful, there are cases in which pain is also elicited along with hip motion, mainly because of inflammation of the trochanteric bursa caused by the ITB sliding on the GT [4- 6]. This painful condition is usually reported as external snapping hip syndrome (ESHS).

According to the current literature, the first-line treatment for ESHS is conservative management with good results for most cases. Non-operative treatment includes rest, non-steroidal anti-inflammatory drugs (NSAIDs), local corticosteroid injections, stretching exercises, physiotherapy, and shockwave therapy [2,4,5]. For persistent or recalcitrant cases after at least three months of conservative management, an operative treatment is indicated. There are many different procedures for ITB lengthening described in the literature, either open or endoscopic. Traditionally, an open Z-plasty or N-plasty of the ITB is performed with reportedly good functional outcome [5]. However, all open techniques are associated with a higher risk of wound complications, hematomas, and slower recovery [3,5,6]. The development of endoscopic procedures has provided a viable solution to the disadvantages of the open techniques. As there is evidence supporting that endoscopic techniques are superior regarding complication rates and patient satisfaction rates, they are gaining popularity among hip surgeons [3,6].

The aim of this study is to assess the clinical outcome of endoscopic ITB release using a modified Ilizaliturri method in seven patients with ESHS and evaluate the safety and effectiveness of our technique.

## Materials and methods

This was a retrospective case series conducted in the following hospitals: European Interbalkan Medical Center, Thessaloniki, Greece and Mediterraneo Hospital, Glyfada, Athens, Greece from January 2021 to September 2024. Seven patients with persistent hip pain due to ESHS were included in this study. Inclusion and exclusion criteria are listed in Table 1. All diagnoses were made after thorough medical history and clinical examination, and confirmed by dynamic hip sonography. Key findings of the clinical examination were a positive Ober's test in most cases and a painful palpable or visible snapping during hip extension and internal rotation. X-rays were routinely performed: anteroposterior and Dunn views. An MRI was also obtained for all patients for more detailed imaging and to exclude other causes of hip pain. All surgeries were performed by the same experienced hip surgeon in the same institution. Concomitant defects were also treated in the same operation. The demographic characteristics and coexisting lesions are provided in Table 2.

**Table 1:** Inclusion and exclusion criteria

Inclusion criteria	Exclusion criteria
Both male and female patients over 18 years of age	Inflammatory or septic arthritis
Unilateral or bilateral painful snapping hip syndrome	Local injection therapy less than three months prior to surgery
Failure of at least six months of conservative treatment with physiotherapy and injections	
Minimum of six months follow-up	

**Table 2:** Demographics and coexisting lesions

Patient No	Age (years)	Sex	Height (m)	Weight (kg)	BMI (kg/m^2^)	Activity	Concomitant lesions
1	19	f	1.65	50	18.4	Ballet, basketball (non-professional)	None
2	28	f	1.70	54	18.7	Basketball (non-professional)	None
3	34	f	1.65	54	19.8	Swimming, pilates (non-professional)	Chodral lesion and subchondral cyst (acetabulum)
4	52	f	1.66	60	21.8	---	Degenerative defects of labrum
5	28	f	1.73	69	23.1	---	Labral tear
6	33	M	1.83	84	25.1	Football (professional)	Degenerative defects of labrum: cam lesion
7	23	F	1.62	53	20.2	Basketball (non-professional)	None
mean values	31		1.69	60.57	21.01		

The modified Harris Hip Score (MHHS) and the Hip Outcome Score (HOS) were implemented for the preoperative and postoperative functional evaluation of the patients [7,8]. These scales are available for use without any permissions needed. The results were obtained by a proxy who directly translated the scores. All data were analysed by an independent statistician. The paired t-test for normally and the Wilcoxon signed-rank test for non-normally distributed data were performed to analyze outcomes between preoperative and postoperative findings, respectively. p-values less than 0.05 were considered statistically significant.

Surgical technique

**Figure 1:**
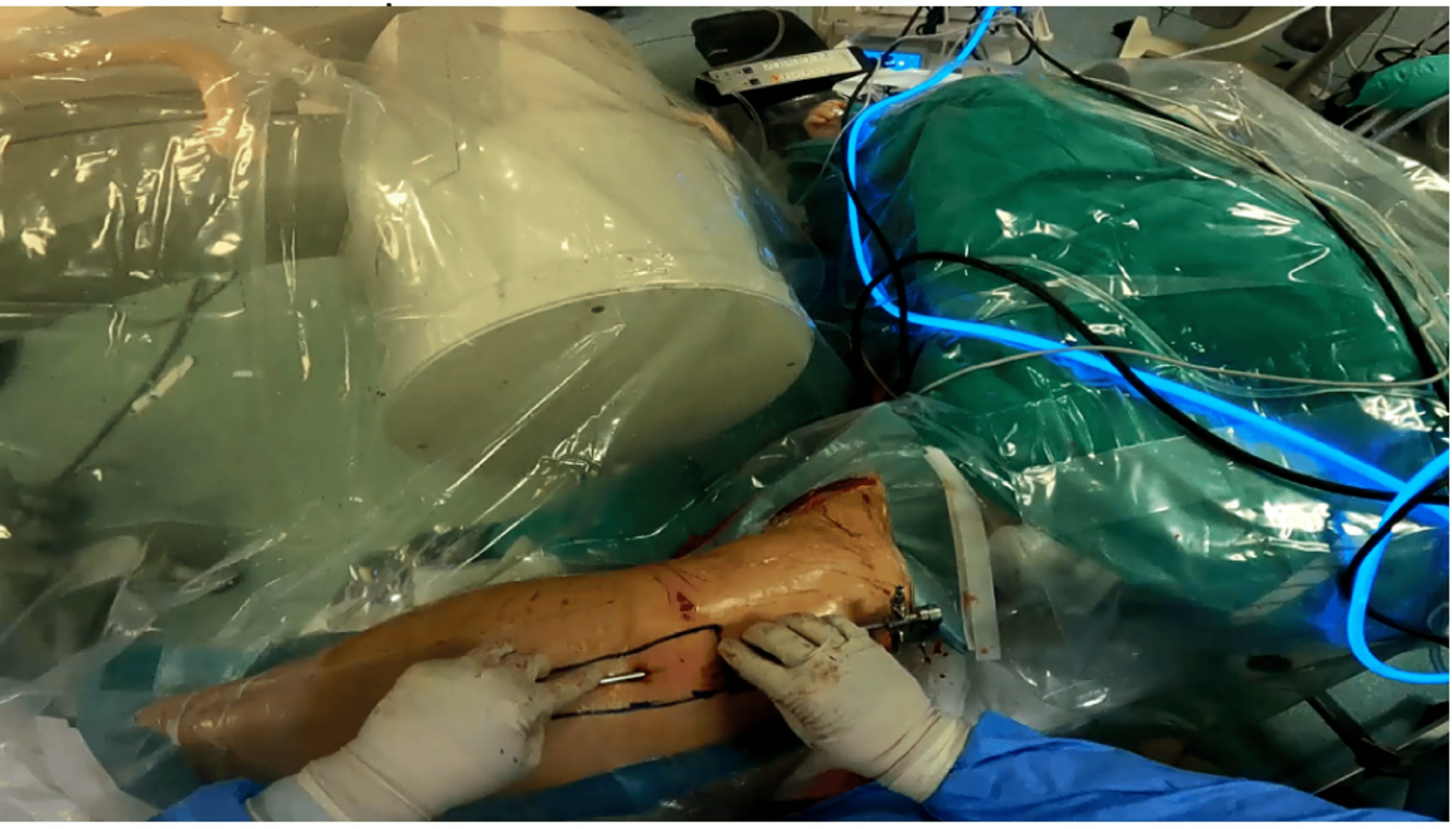
Lateral decubitus placement of the patient and portal establishment under fluoroscopic guidance. The greater trochanter is used as a landmark to establish the superior and inferior portals

The patients were placed in a lateral decubitus position (Figure 1) with the affected limb in a traction system and slightly lateralized with the use of a cylindrical padded post. Hip arthroscopy of the central and peripheral compartments was performed on all patients before endoscopic ITB release, and a coexisting cam lesion was treated in one patient.

**Figure 2:**
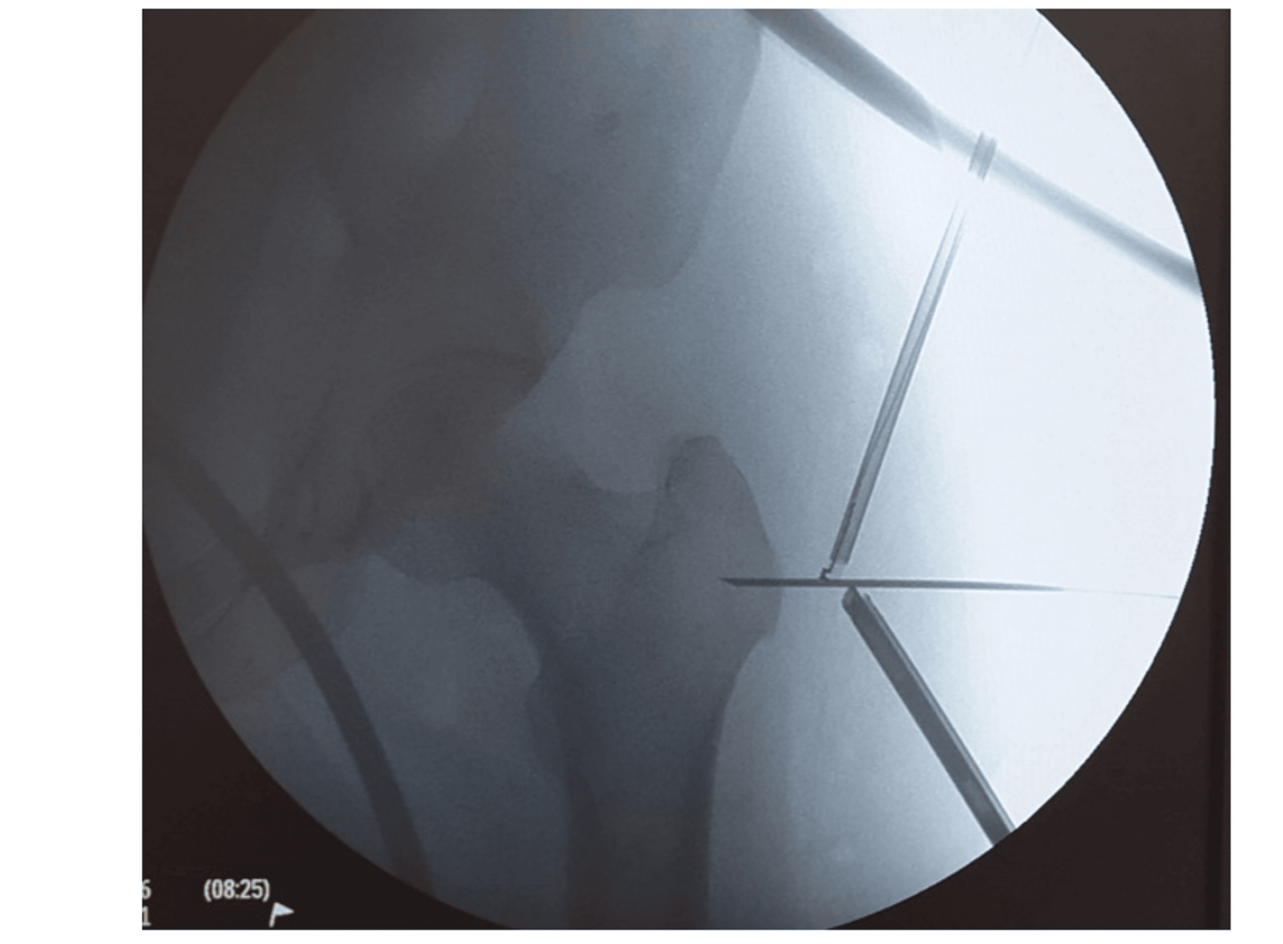
Lateral decubitus placement of the patient and portal establishment under fluoroscopic guidance. The greater trochanter is used as a landmark to establish the superior and inferior portals

**Figure 3:**
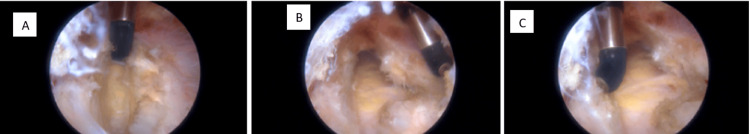
Arthroscopic images of the iliotibial lengthening technique. A vertical incision of the iliotibial band (A) followed by two smaller horizontal cuts on both the anterior (B) and posterior (C) flaps result in a "cross-shaped" defect.

Following arthroscopy, 40-50 ml of saline was introduced with a needle placed under fluoroscopic guidance directly over the greater trochanter to create an open space. After that, using the GT as a landmark lying in the midline of the two portals, the superior trochanteric and inferior trochanteric portals (Figure 2) were established, as described by Ilizaliturri et al. [9]. After blunt dissection of the subcutaneous tissues above the greater trochanter, the ITB was visible with the standard 30-degree, 4-mm scope, and its anterior and posterior borders were confirmed. The gluteus maximus insertion to the ITB was used as an additional landmark for the level of incision at the longitudinal axis. A longitudinal incision was performed starting 4 cm distally from the greater trochanter and directed centrally, towards it. Then, a horizontal cut of 2 cm was made on the anterior flap, vertical to the initial cut, and another horizontal cut was made in the same fashion on the posterior flap (Figure 3). All the incisions of the ITB were made using a radiofrequency ablator. The final result was a cross-shaped cut on the ITB. Unlike the original technique that Ilizaliturri et al. [10] described, no further flap removal was performed on the patients of the present study. Adequate release of the fascia lata and elimination of the snapping was intraoperatively confirmed with passive full range examination of the hip under anesthesia. All patients were discharged on the day of surgery.

Partial weight bearing was permitted for the first six weeks, followed by full weight bearing. Physiotherapy commenced one week postoperatively with aggressive stretching exercises of the ITB to prevent retightening. All patients were routinely followed up postoperatively in two weeks for removal of sutures, and then at three weeks, three months, and six months. For the sake of this study, all the patients were re-evaluated clinically or by telephone conversation.

## Results

Six out of seven patients had an uneventful recovery. A minor complication was found in one patient who presented with direct postoperative diffuse thigh swelling that resolved spontaneously in 24 hours. After six weeks, all patients were able to fully return to their daily activities.

The MHHS and HOS scores were obtained preoperatively and compared with postoperative scores at the final follow-up evaluation, as shown in Table 3. Further analysis revealed a statistically significant improvement in both scores (Table 4). MHHS was increased from 52.33±13.97 (mean ± SD) to 97.27±2.76, which represents a very significant difference (paired samples t-test t (6) = 8.73, p<0.001 or Wilcoxon signed-rank test z (6)=2.37, p=0.018). Accordingly, HOS was improved from 44.99±10.31 (mean ± SD) to 92.85±5.35. This is also a significant difference whether using the paired samples t-test t(6)=13.47, p<0.001 or the Wilcoxon signed-rank test z(6)=2.37, p=0.018.

**Table 3:** Preoperative and postoperative values of HOS and mHHS HOS: Hip Outcome Score, mHHS: modified Harris Hip Score

Patient	Preoperative MHHS	Postoperative MHHS	Preoperative HOS	Postoperative HOS
1	67.1	93.5	46.80%	95.60%
2	51.7	95.7	50%	85.90%
3	64.9	100.1	61.7%	100%
4	46.2	95.7	45.31%	91.20%
5	53.9	100.1	40.47%	98.8%
6	25.3	95.7	27.5%	88.75%
7	57.2	100.1	43.18%	89.77%

**Table 4:** Analysis of preoperative and postoperative values of HOS and mHHS HOS: Hip Outcome Score, mHHS: modified Harris Hip Score

Preoperative MHHS, mean±SD	Postoperative MHHS, mean±SD	t-test	z	Preoperative HOS, mean±SD	Postoperative HOS, mean±SD	t-test	z
52.33±13.97	97.27±2.76	8.73 (p<0.001)	2.37, (p=0.018)	44.99±10.31	92.85±5.35	13.47 (p<0.001)	2.37, (p=0.018)

## Discussion

Painful snapping hip can be a challenging condition for orthopedic surgeons as it affects mainly the young population engaged in high-level physical activity. Although the majority of patients improve with conservative means and physiotherapy, there are persistent cases in which surgical treatment is required [10]. The traditional open procedures that have been described are associated with inconsistent outcomes regarding the elimination of snapping and pain alleviation, and with a relatively high complication rate up to 22% and poor cosmesis due to larger scars [3,5,10,11].

With the advancement of arthroscopy, endoscopic release of the ITB is raising interest among surgeons, and various techniques have been proposed. Endoscopic incision of the ITB complex on different levels and in variable shapes is described and offers safe, aesthetically improved results and relief of the painful snapping hip that allows earlier mobilization and return to activity.

Ilizaliturri et al. described a diamond-shaped ITB release carried out with the radiofrequency probe and shaver, followed by bursectomy in 11 hips [9]. Compared to open procedures, their technique showed excellent functional outcome and relief of pain with a low recurrence rate, and they noted one refractory painless snapping two years after surgery. With the same technique, but performed inside-out, similar findings were reported by Yoon et al. [12]. In their study, including seven patients and 10 hips, no recurrence and no major complications were reported.

Shrestha et al. presented 248 patients with ESHS treated arthroscopically using a single anteroposterior, horizontal cut to release the ITB [5]. This retrospective study reports excellent results regarding the improvement of hip ROM and no major complications, in one of the largest samples found in the current literature. In a study by Zhang et al., a single 5-cm transverse cut of the ITB at the level of the GT was described and showed encouraging results in 16 patients with combined femoroacetabular impingement (FAI) and ESHS [13].

Polesello et al. proposed another endoscopic technique to release tension on the ITB by performing a tenotomy of the femoral insertion of gluteus Maximus [14]. This method was developed to overcome one of the disadvantages of the “ITB defect” techniques, which is the lateral thigh shape deformity. In a series of eight patients (nine hips) treated with the Polesello technique, one required revision surgery due to persistent symptoms, and one reported mild painless snapping. All patients were able to return to their preoperative level of physical activity.

Based on the heterogeneity of the pathophysiology of the ESHS and the different structures that may be involved, Malinowski et al. introduced a “fan-like” technique to gradually reduce the tension of the ITB complex [6]. Under direct inspection with the scope and intraoperative ROM evaluation, subsequent incisions are made to release the affected structures. This endoscopic method provides a relatively simple but more individualized approach depending on the underlying pathology of the ESHS.

While the literature is unclear whether the endoscopic approach is appropriate for revision cases, Mak and Lui have published a technical note on their preferred treatment method for recurrence after endoscopic management of ESHS [15]. They showed that a revision using a combination of the standard Ilizaliturri technique, enhanced with removal of the fibrous tissue and bursa over the GT and gluteus maximus tenotomy, might provide a viable solution that avoids an open procedure.

A number of variations of the above techniques have been proposed in recent publications. Randelli et al. presented a pictorial review of the endoscopic procedures that aim at the elimination of ESHS [2]. Although there is concern over these publications as they encompass mainly small case series, their results are very encouraging. All endoscopic techniques are shown to have a favorable functional outcome with very low complication and recurrence rates compared to the open techniques [2]. Giai Vai et al., in their systematic review, report a recurrence rate as low as 1.02% [3]. In a total of 689 patients, the hip ROM after endoscopic ΙΤΒ release was improved, and also other associated conditions, such as knee pain, were resolved without further interventions. In cases of combined FAI and ESHS, it is suggested that the patient-related outcome is favorable when both entities are addressed in a single endoscopic procedure compared to sole arthroscopic treatment of FAI combined with conservative management of ESHS [13].

Adding to the current literature, the present study demonstrates a simple modification of the standard Ilizaliturri technique, an endoscopic cross-shaped release of the ITB without creating a large diamond-shaped defect that avoids a possible effect on the lateral thigh shape. In conjunction with the removal of the inflamed bursa, the proposed technique can lead to effective relief of the pain and snapping with limited complication risk.

The small sample size and the retrospective design are the main limitations of this study. However, this reflects the rarity of ESHS cases that need to be treated operatively. A further limitation is the heterogeneity of the timing of the postoperative evaluation, as the final follow-up assessment using the functional scores is different for every patient, varying from six months postoperatively to one year. Additional evaluation would be useful to determine the long-term functional outcome in patients who underwent endoscopic ITB release as treatment to ESHS.

## Conclusions

The present study presents a safe and effective endoscopic procedure to manage the ESHS. Following the principles of Ilizaliturri’s technique, an adequate release of the ITB and full resolution of the painful snapping can be obtained with minimal complications and recurrence risk. However, more research is required in order to compare the endoscopic treatment of ESHS to open procedures.
